# Delayed initiation of enteral feeds is associated with postnatal growth failure among preterm infants managed at a rural hospital in Uganda

**DOI:** 10.1186/s12887-020-1986-5

**Published:** 2020-02-24

**Authors:** Clare Nakubulwa, Victor Musiime, Flavia B. Namiiro, James K Tumwine, Christine Hongella, James Nyonyintono, Anna B. Hedstrom, Robert Opoka

**Affiliations:** 1 Department of Pediatrics and Child Health, School of Medicine, Makerere University College of Health Sciences, PO Box 7072, Kampala, Uganda; 2 Survival Pluss Project, Luwero, Uganda; 3 Kiwoko Hospital, P0 Box 149, Luwero, Uganda; 4 University of Washington/Seattle Children’s Hospital, Seattle, USA

**Keywords:** Postnatal nutrition, Enteral feeds, Growth outcomes, postnatal growth failure

## Abstract

**Background:**

Prematurity is the leading cause of mortality in children under 5 years of age globally and is also frequently associated with postnatal growth failure (PGF). Although most preterm births occur in low resource settings, little is known about their postnatal growth outcomes especially in rural areas*.* We evaluated the incidence and factors associated with PGF among preterm infants managed at a rural hospital in Uganda.

**Methods:**

Retrospective cohort study of preterm infants discharged from Kiwoko Hospital neonatal intensive care unit (NICU) from July 2017 to June 2018. Inclusion criteria included gestational age 26 up to but not including 37 weeks, admission within 24 h of birth and at least 7 days hospital stay. Exclusion criteria included major congenital anomalies and missing gestational age or birth weight. Birth and discharge weights from clinical notes were plotted on Fenton 2013 growth charts. Gestation age was determined by last normal menstruation period (LNMP), extracted from the mother’s antenatal card or early obstetric ultrasound scan reports. Postnatal growth failure was diagnosed if discharge weight was less than the 10th percentile for estimated gestational age. Other data from the clinical notes included demographic characteristics, neonatal morbidities as assigned by the attending physician and infant feeding practices. Multivariable logistic regression was used to explore factors associated with PGF.

**Results:**

A total of 349 preterm infants with a mean gestational age of 31 (range 26 to 36) weeks were included. The incidence proportion of PGF was 254/349 (73%). Factors significantly associated with postnatal growth failure included: delayed initiation of enteral feeds [AOR = 3.70, 95% (CI 1.64 to 8.33)], sepsis [AOR = 6.76, 95% (CI 2.15 to 21.2)], multiple gestation [AOR = 1.81, 95% (CI 1.01 to 3.24)] and male gender [AOR = 1.71 95% (CI 1.01 to 2.91)].

**Conclusion:**

Nearly three quarters of preterm infants managed at a rural hospital in Uganda had postnatal growth failure. Delayed initiation of enteral feeds and sepsis were highly associated with postnatal growth failure. Enteral feeds should be initiated as soon as possible in these infants to reduce early protein deficits and hence postnatal growth failure.

## Background

Preterm birth is increasing worldwide, with 60% of these births occurring in middle to low income countries of South Asia and Sub-Saharan Africa. In Uganda, preterm infants make up 14% of all live births and Uganda ranks at 13th out of 184 countries in rates of preterm births [1]. Preterm infants experience a nutritional emergency as they are suddenly removed from a nutrient rich environment in-utero to the extrauterine life where nutrition is harder to initiate and later on maintain. Although postnatal nutrition in preterm infants is aimed at them attaining growth rates that approximate the intrauterine fetal growth rates [2] growth lags are reported among these hospitalized infants worldwide [3–5]. These growth deficits are not only reflected by poor weight gain but also head circumference and length. Weight below the 10th percentile of the expected intrauterine size of fetuses of the same gestational age is termed as postnatal growth failure (PGF) [3].

Early growth deficits amongst preterm infants results from protein and energy deficits after birth [6] and are associated with poor growth and neurodevelopmental outcomes later in childhood [7, 8]. Even with growing evidence that optimizing nutrition with total parenteral nutrition, early enteral feeds with fast advancement and fortification of human milk improves growth outcomes, resource limited settings have not been able to implement all this because of cost. In Uganda, little has been documented concerning growth outcomes of preterm infants especially in rural areas.

We evaluated the incidence and factors associated with postnatal growth failure amongst preterm infants managed at Kiwoko Hospital- a rural district hospital in central Uganda.

## Methods

### Study design

This was a retrospective cohort study using in-patient records of preterm infants for a 1 year period (July, 2017 to June, 2018).

### Study setting

The study was carried out at Kiwoko Hospital, a rural faith-based hospital in Nakaseke district. The neonatal unit opened in 2001 and is supported by Adara Development an international non-governmental organisation. The unit is well-established and recognised in Uganda as a centre of excellence in newborn care. Services are offered at subsided prices to mainly three rural districts of central Uganda; Nakaseke, Luwero, Nakasongola as well as a few cases from other surrounding districts. With an official bed capacity of 38, the unit admits over 100 neonates per month, 1200 per year of which prematurity accounts for 40% of admissions. It is run by a paediatrician supported by one medical officer and a team of nurses. Unit resources include constant electricity, incubators, radiant warmers, intermittent pulse oximetry, phototherapy, intravenous pumps, and continuous positive airway pressure (CPAP). The neonate: nurse ratio per shift is on average 5:1.

On admission to the unit, maternal and infant demographic data are recorded on a specified admission sheet by the admitting nurse who also takes anthropometric measurements. Weights are taken using Seca weighing scales with the infants fully undressed. Gestational age is estimated mostly by LNMP from the mother’s antenatal card, few by first trimester scan and rarely Ballard score done by the admitting doctor. Further assessement is done by both the admitting and unit doctor who makes a clinical diagnosis and recommends appropriate investigations and treatment.

For all infants, intravenous fluids with 10% dextrose at 80-100 ml/kg/day are initiated on admission and electrolytes added on day 3. Each infant is reviewed on a daily basis by the unit doctors and a decisions is made on when to start enteral feeds. Expressed breast milk (EBM) is given initially at 10 - 20 ml/kg/day and increased gradually by 10 - 20 ml/kg/d depending on tolerance by the infant as well as the availability of the milk by the mother. Preterm formula is rarely used when mataernal human milk will not be available. There is no donor milk at the facility. For very low birth weight (VLBW) preterm infants feeding is done by nasogastric tube (NGT). More mature and stable infants may feed via cup or at the breast as soon as possible. For breast feeding infants, fluids are stopped once the neonate is breast feeding well and the mother has enough breast milk. For those feeding by cup or nasogastric tube fluids are stopped once the infant can tolerate 15 ml per feed or 150 ml/kg/day whichever comes first. The mother works closely with the nurses on duty to ensure she gives the prescribed feeds and report any feeds intolerence. This information is recorded in a specified feeds monitoring chart kept in the patients’ file and is reviewed daily by the clinician. Neither fortification of human milk nor total parenteral feeding is available at the unit.

Weights are taken on alternate days and feeds are adjusted according to the weight once the neonate starts gaining weight. Supplementation with multivitamins (Grovit drops) and iron (Haemoforte syrup) is started once the neonate is on full enteral feeds. The VLBW and extremely low birth weight (ELBW) infants are discharged once they attain 1.5 kg, with no recorded apneic spells in the past 5 days and have stable vitals in a cot. On discharge, their corrected gestation in weeks, discharge weight and head circumference are recorded.

Files of discharged preterm infants are stored in the records department and data on demographics and clinical variables stored electronically for quality improvement use.

### Study population

Neonates with a gestation age between 26 and 36 completed weeks and 6 days (by LNMP, first trimester ultrasound scan or Ballard score) who were admitted to the unit within 24 h of life and spent 7 or more days in hospital were included into the study. Preterms with major congenital abnormalities and those with missing birth weight or recorded gestation age were excluded from the study. The former were excluded as they were expected to have PGF from other abnormalities.

### Study procedure

Using a structured and pretested data collection tool, we obtained demographic, nutritional and clinical data relevant to the study from patient files that fulfilled the inclusion criteria. This information included: 1) *Perinatal factors*: Date and time of birth, date of admission, estimated gestational age at birth (LNMP/ early obstetric ultrasound scan/ Ballard score), birth weight, sex, discharge weight. Preterm infants were classified as: extremely preterm (< 28 weeks), very preterm (28 to 32 weeks) and moderate to late preterm (> 32 to < 37 weeks) [3].

Maternal characteristics: Age, parity, morbidities like hypertension (HTN) and HIV sero status. 2) *Morbidity during hospitalization:* Need for CPAP, sepsis or necrotizing enterocolitis (NEC) and oxygen requirement after 28 days. These were diagnoses made by the attending clinician and extracted from patient files. 

Definitions of neonatal morbidities in this study were as follows;

- Need for CPAP. Preterm infants with a respiratory severity score of 5 and above (using the Silverman-Anderson score) were started on CPAP. Chest X-rays are not routinely performed. CPAP is the treatment modality used for RDS at the unit sincce surfactant and ventilators are not available.
- Sepsis: Clinical diagnosis with abnormal axillary temperature (> 38.5° or < 36 °C), fast respirations (above 60 breaths per minute) with refusal to breastfeed or new apnoea/ oxygen requirement in one who was previously stable and in the absence of anaemia. Blood cultures are not available.
- PDA: A continuous murmur most prominent at the upper left sterna border in a preterm infant with significant cardiovascular compromise (tachycardia, tachypnea).
- NEC: Increased gastric residues/ emesis/ bloody stools with abdominal distention, unstable vitals (tempereature, respiration, heart rate) plus or minus abnormal abdominal radiographs.
- Apnea: Cessation of breathing for 20 s or longer, or a shorter respiratory pause associated with bradycardia and desaturations.
- Anaemia: Haemoglobin count of < 8 g/dL if asymptomatic and off oxygen, < 10g/dL if on oxygen, or < 12g/dL if having apnoeas.

3*) Nutrition information:* Dates and time of initiating enteral feeds, any documented feeds intolerance (increased gastric residue with or without vomiting of feeds) and date of attaining full enteral feeds. Delayed initiation of enteral feeding in this study was if feeds were started after 48 h of life.

Birth and discharge weight for each preterm were plotted on Fenton growth charts 2013 [9, 10] and the percentile for each weight read. We used the different charts for boys and girls. We used these charts because they were based on a meta-analysis with the highest number of newborns to date [2]. Birth weight less than the 10th percentile for the estimated gestation age and sex was categorized as small for gestation age (SGA) and that between the 10th and 90th percentile as appropriate for age (AGA). Postnatal growth failure was defined as discharge weight less than the 10th percentile of the expected intrauterine growth for the same postmenstrual age (PMA) and sex [3]. Discharge in this study included those who went home alive as well as those who died after being hospitalized for seven or more days.

### Statistical analysis

Statistical data analysis was done using STATA version 14 (College station, TX). Continuous data were expressed as mean and standard deviation if normally distributed, median and interquartile range if non-normal. Categorical data were expressed as frequencies and proportions. We used Chi square and Fischer’s Exact test to test for presence of association between PGF and the categorical independent variables. The Student’s t- test was used for normally distributed continuous variables and Mann-Whitney U test for those with a skewed distribution.

The cumulative incidence of PGF among preterm infants was determined as the number of new cases of PGF out of the total number of infants at risk in the sample.

We used binary logistic regression to find out the association between the independent variables with PGF. Factors that achieved a *p*-value of less than 0.25 at bivariable analysis and those with biological plausability including gestational age and maternal morbidities (HIV and hypertension) were inserted into a multivariable logistic model. Hosmer-Lemeshow test was used to test the goodness of fit for the model. Adjusted odds ratios and their 95% confidence intervals were obtained to assess for strength of association and the level of significance was set at 5%.

## Results

A total of 494 preterms were admitted to the unit from July 2017 to June 2018. Of these 130 didn’t meet the inclusion criteria and 15 had exclusion criteria. This left a total of 349 preterm infants with a mean gestational age of 31 (range 26 to 36) weeks that were eligible and these were included into the study. The 130 preterm infants who did not meet inclusion criteria included 40 pretems who died within the first week of life, 88 who were discharged before 7 days of life and 2 was was transferred to other units. The 15 preterm infants meeting the exclusion criteria included 5 who had congenital abnormalities, 7 with missing records of gestation age or birth weight, 2 admitted at 2 weeks of age and 1 who was born to a diabetic mother.

### Baseline characteristics

One hundred eighty eight preterm infants were female (53.9%). Majority were moderate to late preterms 167 (47.9%) and very preterm 168 (48.1%). The median length of hospital stay was 14 days (interquatile range (IQR) =23). Majority of the mothers were aged 20–24 (33%), 106 (30.5%) mothers had a parity of one and the prevalence of HIV among the mothers was 8.3% (Table 1).

**Table 1:** Baseline characteristics of the sample population

Baseline Characteristics	Median (min-max)	Number(N)
		n(%)
		*N* = 349
Infant Characteristics		
Sex of infant		
Female		188 (53.9)
Male		161 (46.1)
Gestational age in weeks		
Early preterm	27 (26–27)	14 (4.0)
Preterm	30.5 (28–32)	168 (48.1)
Late preterm	34 (33–36)	167 (47.9)
Growthcategorization at birth		
SGA		36 (10.3)
AGA		313 (89.7)
Birth weight categories		
ELBW	0.96 (0.87–0.99)	18 (5.2)
VLBW	1.26 (1–1.49)	106 (30.4)
LBW	1.84 (1.5–2.8)	225 (64.5)
Length of hospital stay (days)	14 (7–85)	
Discharge status		
Alive		333 (95.42)
Singleton/Multiple birth		
Multiple		122 (35)
Place of birth		
In-born		187 (53.6)
Mode of delivery		
SVD		272 (77.9)
Maternal Characteristics		
Maternal age category^a^		
≤ 19		77 (22.1)
20–24		114 (32.7)
25–29		89 (25.5)
30–34		38 (10.9)
≥ 35		26 (7.4)
Missing data		*5 (1.4)*
Parity categories^a^		
Para1		106 (30.4)
Para2		81 (23.2)
Para3		52 (14.9)
Para4 and above		109 (31.2)
Missing data		*1 (0.29)*
Residence^a^		
Luwero		162 (46.4)
Nakaseke		103 (29.5)
Nakasongola		61 (17.5)
Others		22 (6.3)
Missing data		*1 (0.28)*
Maternal HIV seropositivity		
Yes		29 (8.3)
Maternal Hypertension		
Yes		23 (6.6)
Maternal Diabetes mellitus		
Yes		1 (0.3)

*N* = 349 however for ^a^ where we had some missing data. *AGA* Appropriate for age, *ELBW* Extremely low birth weight, *C/S* Caesarean section, *LBW* Low birth weight, *SGA* Small for gestational age, *SVD* Spontaneous vertex delivery, *VLBW* Very low birth weight

The prevalent common morbidity during hospitalization was Respiratory Distress Syndrome (RDS) with 121/349 (34.7%) requiring CPAP and 47/349 (13.5%) were managed for sepsis. Enteral feeds were initiated within the first 48 h in 80.1% of these preterm infants (Table 2).

**Table 2:** Clinical and nutrition characteristics of the preterm infants

Variables	Median (min -max)	Frequency
		n(%)
Clinical Characteristics		
CPAP		
Yes		121 (34.7)
CPAP duration (days) median (iqr)		4 (5)
Apnea		
Yes		29 (8.3)
Sepsis		
Yes		47 (13.5)
NEC		
Yes		10 (2.9)
PDA		
Yes		11 (3.2)
Requiring O2 ≥ 28 days		
Yes		6 (1.7)
Anemia		
Yes		51 (14.6)
Route of initial feeds^a^		
BF		122 (35.0)
NGT		113 (32.4)
Cup		110 (31.5)
Missing data		*4 (1.14*)
Time of initiating enteral feeds^a^		
> 48 h		69 (19.8)
< 48 h		277 (79.4)
Missing data		*3 (0.86)*
Days until initiating enteral feeds		
Overall	1 (0–7)	
> 48 h	3 (2–7)	
< 48 h	1 (0–2)	
Feeds intolerance		
Yes		28 (8)

*CPAP* Continuous positive airway pressure, *iqr* Interquartile range, *NEC* Necrotizing enterocolitis, *PDA* Patent ductus arteriousus, *NGT* Nasal gastric tube, *N* = 349 ^a^ indicates where we had some missing data

**Fig. 1:**
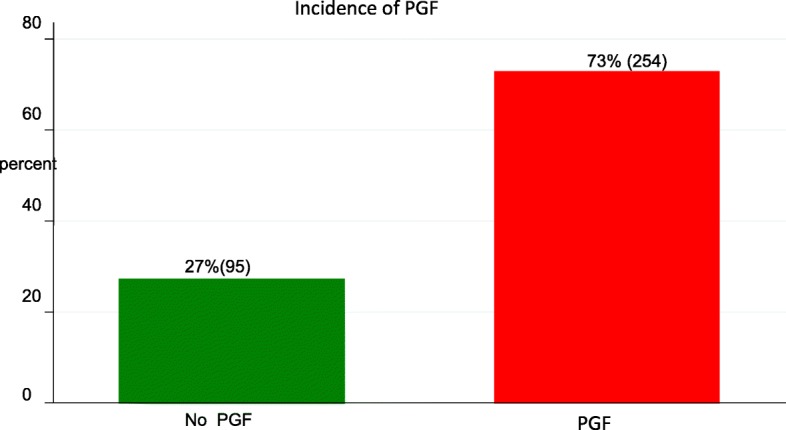
Bar graph showing the incidence of post-natal growth failure among preterm infants managed at Kiwoko Hospital

**Fig. 2:**
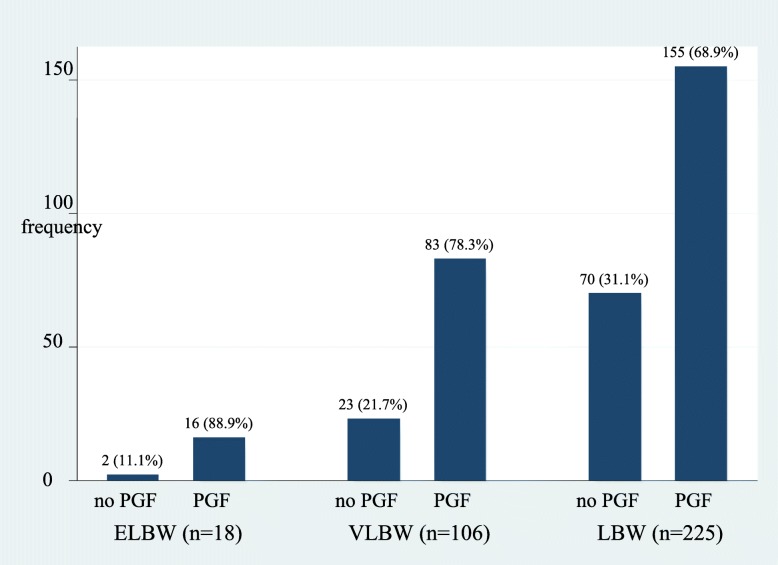
Bar graph showing the incidence of postnatal growth failure by birth weight category. This figure demonstrates the incidence of PGF increased with decreasing birth weight

In this study 73% (254/349) preterm infants had PGF at discharge (Fig. 1) and the incidence increased with decreasing gestation age as shown in (Fig. 2).

Factors associated with PGF at bivariable analysis are shown in Table 3 and at multivariable analysis, delayed initiation of enteral feeds [AOR = 3.70, (95% (CI 1.64 to 8.33)], sepsis [AOR = 6.76, (95% (CI 2.15 to 21.2)], multiple gestation [AOR = 1.81, (95% (CI 1.01 to 3.24)] and male gender [AOR = 1.71 (95% (CI 1.01 to 2.91)] were significant factors. On the contrary, gestational age, maternal age, and HIV status were not found to be significantly associated with the occurance of PGF (Table 4).

**Table 3:** Bivariable analysis for factors associated with extrauterine growth restriction among preterm infants managed at Kiwoko Hospital

Bivariable analysis		
Variable	OR(95%CI)	*p*-value
Sex		
Female	1	
Male	1.59 (0.98,2.57)	0.06
Gestational age Category		
Early preterm	1	
Preterm	0.8 (0.24,2.66)	0.716
Late preterm	1.51 (0.45,5.1)	0.508
Length of hospital stay		
< 14 days	1	
14 days or more	1.04 (1.02,1.06)	**< 0.001**
Birth weight by GA		
SGA	1	
AGA	0.07 (0.01 0.49)	**0.008**
Singleton/Multiple birth		
Singleton	1	
Multiple	1.61 (0.96,2.71)	0.07
CPAP		
No	1	
Yes	2.12 (1.24,3.63)	**0.006**
Apnea		
No	1	
Yes	11.65 (1.56,86.84)	**0.017**
Sepsis		
No	1	
Yes	4.64 (1.62,13.3)	**0.004**
Anemia		
No	1	
Yes	2.22 (1,4.91)	0.05
Time to initiating Oral feeds		
> 48 h	1	
< 48 h	0.29 (0.13,0.64)	**0.002**
Days to full feeds		
< 5 inclusive	1	
Above 5	1.95 (1.17,3.25)	**0.01**
Feeds intolerance		
No	1	
Yes	5.3 (1.23,22.79)	**0.025**

**Table 4:** Multivariable analysis for factors associated with extrauterine growth restriction among preterm infants managed at Kiwoko Hospital

Simple Logistic	Crude OR(95% CI)	*P*-value	Adjusted OR(95% CI)	*P*-value
Sex				
Female				
Male	1.59 (0.98,2.57)	0.06	1.71 (1.01, 2.91)	0.043
Gestation age				
Early preterm	1		1	
Preterm	0.8 (0.24,2.66)	0.716	1.7 (0.4, 7.25)	0.475
Late preterm	1.51 (0.45,5.1)	0.508	4.15 (0.94, 18.43)	0.061
Birth weight				
ELBW	18 (5.2)	0.062	–	–
VLBW	106 (30.4)		–	–
LBW	225 (64.5)		–	–
Birth weight for gestation age				
SGA	1		1	
AGA	0.07 (0.01, 0.49)	0.008	0.12 (0.02, 0.9)	**0.04**
Singleton/Multiple birth				
Singleton	1		1	
Multiple	1.61 (0.96,2.71)	0.07	1.81 (1.01, 3.24)	**0.041**
Sepsis				
No	1		1	
Yes	4.64 (1.62,13.3)	0.004	6.79 (2.15, 21.2)	**0.001**
Time of initiating Oral feeds				
Less than 48 h	1		1	
More than 48 h	3.44 (1.56, 7,69)	0.002	3.70 (1.64, 8.33)	**0.002**
HIV				
No	1		1	
Yes	1.88 (0.7,5.07)	0.214	1.82 (0.63, 5.26)	0.272
Hypertension				
No	1		1	
Yes	2.62 (0.76,9.03)	0.127	1.67 (0.45, 6.17)	0.443

## Discussion

We evaluated the incidence of and factors associated with PGF in hospitalized preterm infants in a neonatal care unit in a rural population in Uganda. We found a very high incidence of PGF- 73% of the preterm infants were below the 10th percentile for weight at discharge. PGF was not only found in the extremely preterm infants but also in the moderate to late preterms. The findings are consistent with other studies that show that PGF is common in hospitalized preterm infants worldwide. Kumar et al. in a prospective observational study in a level III NICU at a tertiary care teaching hospital in India found the incidence of PGF to be 64.8% from a study population of 111 preterm infants [4]. Clark et al. reported 28% PGR among preterm infants who were 23 to 34 weeks in a multi-center study in USA [3]. A more recent study in Korea found postnatal growth failureof 45.9% from a sample of 2799 VLBW preterms [5]. In Africa, a study done in South Africa also demonstrated early growth failure in a cohort of VLBW preterm infants [11]. The variation in frequency of PGF in these studies can in part be explained by different demographic characteristics, cut offs to define PGF, unit resources and the use of different feeding protocols.

Poor post-natal growth (weight, length or head circumference) is primarily due to inadequate nutritional intake [6]. This early growth deficit affects brain development and has been shown to result in poorer neurodevelopmental outcomes later in life [7, 8]. 

This study showed that delayed initiation of enteral feeds that is after 48 h of life was 3.7 times more associated with PGF compared to when feeds were started within the first 48 h of life. A South African study on growth of 92 extremely low birth weight preterm infants at a tertiary hospital also found that being kept nil per os beyond the first day of life was significantly associated with poor growth [12]. Earlier enteral feeding results in earlier achievement of full enteral feeds which means reduced cumulative protein and energy deficits and therefore more weight gain [13]. In low resource settings where parenteral nutrition is not widely used, earlier initiation of enteral feeds may best optimize postnatal nutrition. The WHO also recommends breast feeding within an hour of birth as well as minimal enteral feeds on day one of life for those unable to breastfeed [14].

Although the American Academy of Paediatrics suggests that postnatal growth in preterms should mimic their intrauterine foetal growth rates at the same estimated gestational age, this growth trajectory was not reflected in this study population. Embleton et al. in 2001 related this growth lag to the cumulative energy and protein deficits that occur in the postnatal period [6]. It takes time to initiate as well as maintain adequate nutrient intake in preterm infants and some studies concluded that PGF in preterms is inevitable. Emerging evidence, however, shows that optimizing nutrition can improve post-natal growth in preterms and reduce the incidence of PGF [15, 16]. A study by Young et al on 124 infants in Korea reported that day 1 parenteral feeding with greater supply of protein and energy decreased the incidence of PGF in VLBW preterms [16]. Another study on 396 preterm infants below 32 weeks gestational age at a university hospital in UK further demonstrated that early postnatal growth failure is not inevitable. The study showed that with early enteral feeding, fortification of human milk as well as employing a multidisciplinary nutrition team in the neonatal unit, infants grew at rates comparable to their intrauterine rates [17].

In this study, preterm infants with sepsis were 6.76 more times likely to have PGF compared to those without sepsis. Being ill increases the basal metabolic requirements and yet the sick preterms are in most cases fed less due to concerns of  instability and ability to digest feeds. This creates protein and energy deficits consequently leading to poor growth as has been reported by Bertino et al. 2006 in Italy, Lee et al. 2018 in Korea, and Mabhandi et al. 2019 in South Africa [5, 12, 18].

The incidence of PGF has been shown in some studies to increase as gestational age and birth weight decrease because smaller preterm infants lose more weight and take longer to regain it. Also as gestation age decreases, nutritional requirements increase and achieving satisfactory growth rate become less likely [3, 19]. In as much as our study was able to demonstrate an increase in the incidence of PGF with decreasing birth weight (Fig. 2), the incidence was highest in moderate to late preterms compared to extremely preterm infants (79% Vs 71.4%) at univariate level. Shan et al. [20] and Freitas et al. [21] associated PGF with increasing gestational age which is consistent with our findings. These studies like ours included all preterms less than 37 weeks but earlier studies focused on preterm infants less than 34 weeks and could have missed the growth dynamics in older preterms. At multivariable analysis gestational age was not significantly associated with PGF in this study.

This study had a number of limitations. Given that it was a retrospective chart review, there was missing information from the files that resulted in some patient files being excluded from the study. Weight measurements are subject to errors and some may been incorrectly done leading to misclassification. LNMP was the most commonly used method for estimating gestational age in this population and is less precise than a first trimester ultra sound scan. We were unable to quantify the actual nutrition intake for these preterms using patient files.

The major strength of this study was the good quality data, carefully recorded weights and patient information in a reasonably well organized model neonatal unit in rural Uganda that enabled us to compile this data and report an important topic. To our knowledge, this was the first study in Uganda to investigate PGF among preterm infants.

## Conclusion

Nearly three quarters of preterm infants managed at a rural hospital in Uganda had postnatal growth failure. Initiating enteral feeds after 48 h of life and sepsis during hospitalization were significantly associated with PGF. Enteral feeds should be initiated as soon as possible in these infants to reduce early protein deficits and hence postnatal growth failure.

## Data Availability

The datasets used and/or analysed during the current study are available from the corresponding author on reasonable request.

## Abbreviations

AGA: Appropriate for gestational age
HIV: Human Immunodeficiency Virus
LBW: Low birth weight
LNMP: Last Normal Menstrual Period
NEC: Necrotizing enterocolitis
NICU: Neonatal Intensive Care Unit
PGF: Postnatal growth failure
SGA: Small for gestation age
VLBW: Very low birth weight
WHO: World Health Organization
